# Supplementary material for: Comparison of Serum and Urine as Sources of miRNA Markers for the Detection of Ovarian Cancer
Source: Biomedicines. 2023 Sep 11;11(9):2508. doi: 10.3390/biomedicines11092508 (PMC10525629; doi:10.3390/biomedicines11092508)
Supplement: Supplementary file 1 [file biomedicines-11-02508-s001.zip › biomedicines-2563495-supplementary.pdf]

biomedicines

MDPI

Supplementary Materials

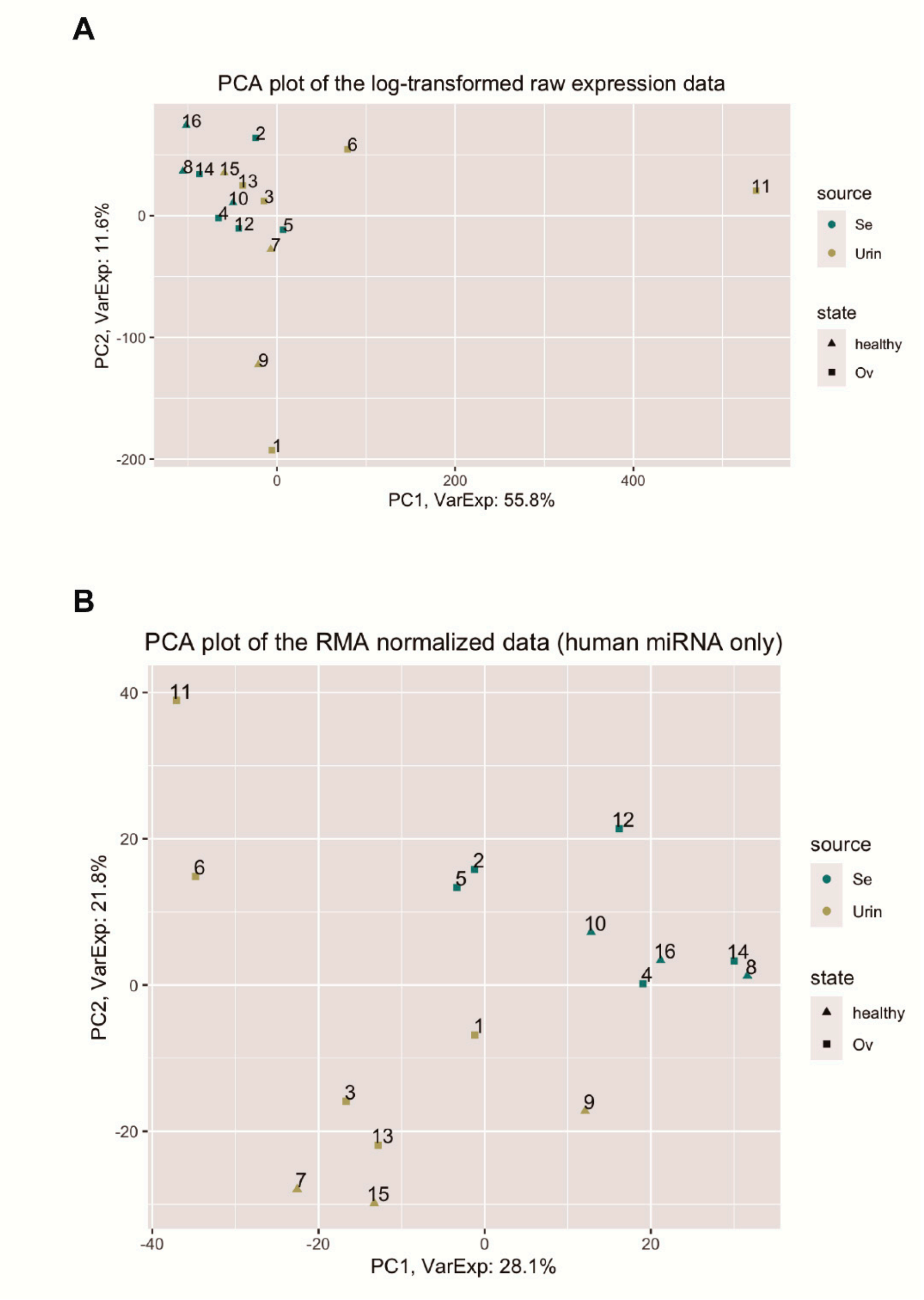

Supplemental Figure S1. Principal component analysis (PCA) of raw and RMA-normalized processed data on the microarray. (**A**) Plot of the PCA of log-transformed raw expression data. (**B**) Plot of the PCA of Robust Multichip Average (RMA) normalized data.

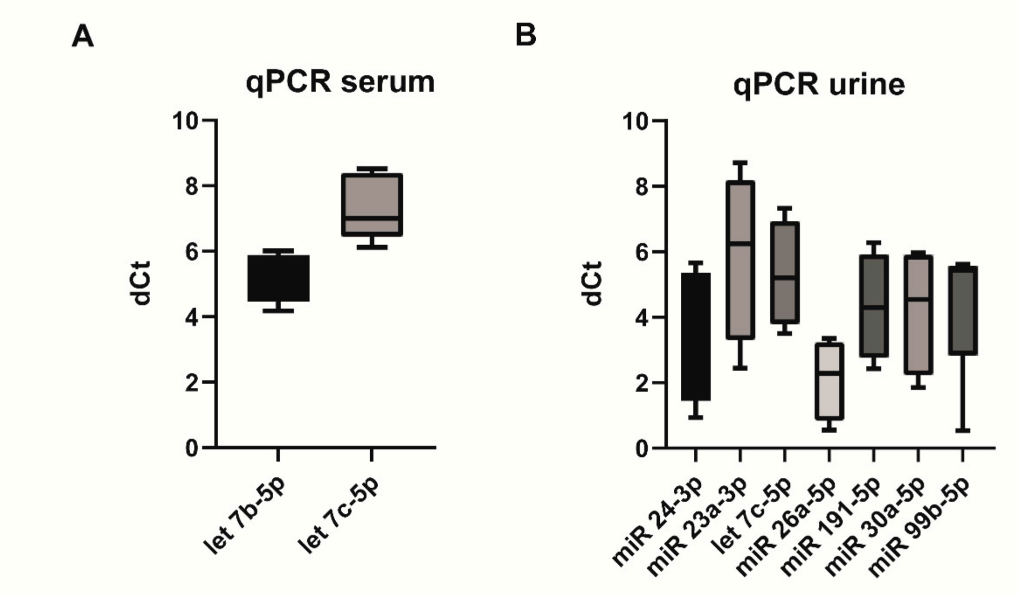

Supplemental Figure S2. Validation of miRNA markers by qPCR. qPCR analysis of chosen miRNAs in serum in patients with ovarian cancer (**A**) in urine in patients with ovarian cancer (**B**).
